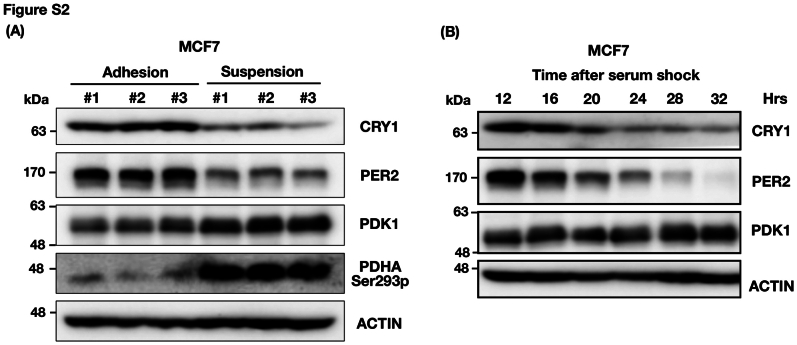# Circadian modulation of glucose utilization *via* CRY1-mediated repression of Pdk1 expression

**DOI:** 10.1016/j.jbc.2024.105637

**Published:** 2024-01-08

**Authors:** Yi-Ying Chiou, Cing-Yun Lee, Hao-Wei Yang, Wei-Cheng Cheng, Kun-Da Ji

**Affiliations:** Graduate Institute of Biochemistry, College of Life Sciences, National Chung Hsing University, Taichung, Taiwan

**Keywords:** circadian clock, cryptochrome, glycolysis, pyruvate dehydrogenase kinase, gene transcription

## Abstract

Life adapts to daily environmental changes through circadian rhythms, exhibiting spontaneous oscillations of biological processes. These daily functional oscillations must match the metabolic requirements responding to the time of the day. We focus on the molecular mechanism of how the circadian clock regulates glucose, the primary resource for energy production and other biosynthetic pathways. The complex regulation of the circadian rhythm includes many proteins that control this process at the transcriptional and translational levels and by protein-protein interactions. We have investigated the action of one of these proteins, cryptochrome (CRY), whose elevated mRNA and protein levels repress the function of an activator in the transcription-translation feedback loop, and this activator causes elevated Cry1 mRNA. We used a genome-edited cell line model to investigate downstream genes affected explicitly by the repressor CRY. We found that CRY can repress glycolytic genes, particularly that of the gatekeeper, pyruvate dehydrogenase kinase 1 (Pdk1), decreasing lactate accumulation and glucose utilization. CRY1-mediated decrease of Pdk1 expression can also be observed in a breast cancer cell line MDA-MB-231, whose glycolysis is associated with Pdk1 expression. We also found that exogenous expression of CRY1 in the MDA-MB-231 decreases glucose usage and growth rate. Furthermore, reduced CRY1 levels and the increased phosphorylation of PDK1 substrate were observed when cells were grown in suspension compared to cells grown in adhesion. Our data supports a model that the transcription-translation feedback loop can regulate the glucose metabolic pathway through Pdk1 gene expression according to the time of the day.

Circadian rhythm is a mechanism to adapt to the daily variation of the environment. Cells regularly vary the strength in their response to many biological processes by controlling the expression of genes following a 24-h cycle (1). The transcription-translation feedback loop (TTFL) is the core mechanism for maintaining the rhythmicity of the cells (2). A complex suit of proteins interacts and influences successful responses to rhythmic environmental changes. In mammals, TTFL is accomplished *via* proteins, circadian locomotor output cycles kaput (CLOCK) protein and brain and muscle ARNT-like 1 (BMAL1) protein, which form a heterodimer (3) to activate the transcription of primary clock-controlled genes (CCGs) by binding the E-box of their promoters (4). Some proteins encoded by these primary CCGs could negatively regulate CLOCK-BMAL1 with some time delay to form the feedback loop (5). Cryptochrome proteins (CRY1 and CRY2) could repress CLOCK-BMAL1-mediated transcription by forming CRY-CLOCK-BMAL1 complex on E-box (6, 7). Period proteins (PER1, PER2, and PER3) could interfere with CLOCK-BMAL1-mediated transcription by displacing CLOCK-BMAL1 from promoters through interacting with CRY proteins and recruiting casein kinase 1 delta to the CLOCK-BMAL1 (6, 7, 8, 9, 10). Nuclear receptor superfamily 1 group D (NR1D) proteins (NR1D1 and NR1D2) could repress the transcription of genes encoding BMAL1 and CRY1 to support the TTFL efficiently (11). Other products of primary CCGs include transcription factors controlling the rhythmic expression of other circadian genes and proteins involved in various cellular functions (12, 13).

Unlike the CRY protein in *drosophila* as a light sensor (14), the ability of mammalian CRY proteins to inhibit CLOCK-BMAL1 is light-independent, supported by the change of circadian patterns of CRY-deficient mice in the all-dark condition and the light-independent transcriptional repression of CRY proteins using reporter gene assays (15, 16, 17). CRY1 and CRY2 have partially overlapped functions in the maintenance of circadian rhythm, supported by the finding that Cry1 or Cry2 KO mice do not entirely lose the circadian rhythm but display distinct phenotypes (15, 16, 18, 19). In the circadian-synchronized cell model, CRY1 is a more potent repressor of CLOCK-BMAL1-mediated transcription than CRY2 and is more powerful in maintaining cell-autonomous circadian rhythm (20). CRY1 or CRY2-specific functions have also been reported to play a role in processes other than the regulation of circadian rhythm (21, 22).

Metabolic and functional oscillations within cells need to be matched. Thus, understanding whether and how the circadian clock regulates cellular metabolism is critical. Glucose is an essential resource of both energy and carbon. Glucose can be converted into pyruvate by glycolysis with ATP production. The fate of pyruvate is either conversion into lactate with the regeneration of NAD^+^ required in glycolysis or conversion into acetyl-CoA for the citric acid cycle (also called the tricarboxylic acid cycle or the TCA cycle). By spending the NADH or FADH_2_ produced from the TCA cycle, oxidative phosphorylation in mitochondria produces more ATP compared to that during glycolysis. Cells modulate the balance of glucose metabolism through glycolysis or the TCA cycle based on changes in physiological circumstances. For example, glycolysis increases when the oxygen concentration decreases (anaerobic glycolysis). Previous research also found that cells with higher proliferation rates utilize more glycolysis at normal oxygen levels (aerobic glycolysis) (23). Thus, it is highly possible that the glucose metabolic pathway within cells is in response to the time of the day.

The core clock, the TTFL in cells, is required to generate peripheral clocks, modulating various physiological functions in different organs, and the master clock in the brain's suprachiasmatic nucleus, coordinating circadian behavior (24). Transcriptional oscillation is expected to be associated with the functional oscillation. Thus, identifying circadian genes from organs provides information about physiological functions modulated by the time of the day. Genes with circadian expression patterns have been analyzed using RNA sequencing analysis of transcriptomes from mouse organs at different circadian times (1). As a result, many genes (hundreds to thousands) were identified that responded in their expression pattern to the different times. Organ-specificity was also observed. Furthermore, although identifying circadian genes from isolated organs provides important physiological implications, it cannot give enough information to understand the regulatory mechanism of these genes. In contrast, transcriptome analysis of an established synchronized cell line model allows the identification of a limited number of circadian genes belonging to the core clock genes, like Per and Per2, which have strong oscillating amplitudes (25) possibly due to gradual desynchronization over time. Cell lines derived from the same origin but mimicking different statuses of the TTFL could be used to study genes controlled by the core clock. The established cell line (CPN^−^^/^^−^) lacking CRY, PER, and NR1D proteins in the negative arms of the TTFL (26) could mimic the status when CLOCK-BMAL1 activity is high. Expression of CRY protein in this cell line mimicking the low CLOCK-BMAL1 activity can study CRY-regulated genes without interference from PER and the feedback loop.

The influence of the TTFL on glycolysis might occur through the hypoxia-inducing factor alpha (HIF1α) pathway. Under hypoxic conditions, HIF1α is stabilized and activates the transcription of lactate dehydrogenase A (Ldha) and pyruvate dehydrogenase kinase 1 (Pdk1) genes (27). LDHA protein is involved in the conversion between pyruvate and lactate, and PDK1 protein inhibits the conversion of pyruvate into acetyl-CoA by phosphorylating pyruvate dehydrogenase (PDH). Increased LDH activity through increased LDHA and decreased PDH activity through phosphorylation by PDK1 shift the metabolic pathway from the TCA cycle to glycolysis. Previous research pointed out that the CLOCK-BMAL1 binds to the promoter of Hif1α and activates the transcription of Hif1α (28). In addition, CLOCK, BMAL1, HIF1α, and HIF1β belong to the basic helix-loop-helix - PER-ARNT-SIM (bHLH-PAS) transcription factor family (29). BMAL1 has been reported to form a complex with HIF1α to activate CLOCK-BMAL1-controlled and HIF1α-HIF1β-controlled genes (30). CRY1 protein has also been implicated in acting as a repressor of HIF1α-HIF1β-controlled genes by interacting with HIF1α (21). Besides regulating the HIF1α protein, the TTFL might regulate hypoxia responses by binding CLOCK-BMAL1 to the promoter regions of HIF1α-HIF1β-controlled genes (28, 31). According to these studies, CLOCK-BMAL1 can bind about 20 percent of the HIF1α binding sites (28), and about 20 percent of the hypoxia-responsive genes showed time-specific effects (31). Thus, although the TTFL could affect the HIF1α protein and HIF1α could affect glycolysis, it is unknown whether the TTFL could regulate glycolysis, especially under conditions with normal oxygen concentration.

In this study, we used a CRY-restituted mouse embryonic fibroblast system to dissect the various conditions involved in circadian rhythm with a particular focus on CRY proteins. We found that CRY protein can decrease lactate production in this cell line system. Using mRNA sequencing, we found genes for glycolysis and lactate production were downregulated by CRY protein expression. We found that CRY1 can regulate the expression of Ldha and Pdk1 by binding to the promoter through CLOCK-BMAL1. To investigate whether CRY1-mediated inhibition of glycolysis could be achieved in different cells, we selected a breast cancer cell line MDA-MB-231 in which glycolysis and its stem cell properties were associated with the PDK1 level (32) and generated a cell line expressing exogenous CRY1 constitutively. CRY1-mediated repression of Pdk1 expression can be observed in the MDA-MB-231 cells. Furthermore, CRY1 expression can decrease the growth rate of MDA-MB-231 cells in adhesion culture and cell aggregation in suspension culture. Our data suggest that the core clock protein CRY1, and thus the TTFL, could control glucose metabolism in cells under normal oxygen concentration. Finally, we present a model to explain our finding on how TTFL controls the cellular glucose metabolism in response to the time of the day.

## Results

### CRY protein decreased lactate production in mouse embryonic fibroblast cells

**Figure 1:**
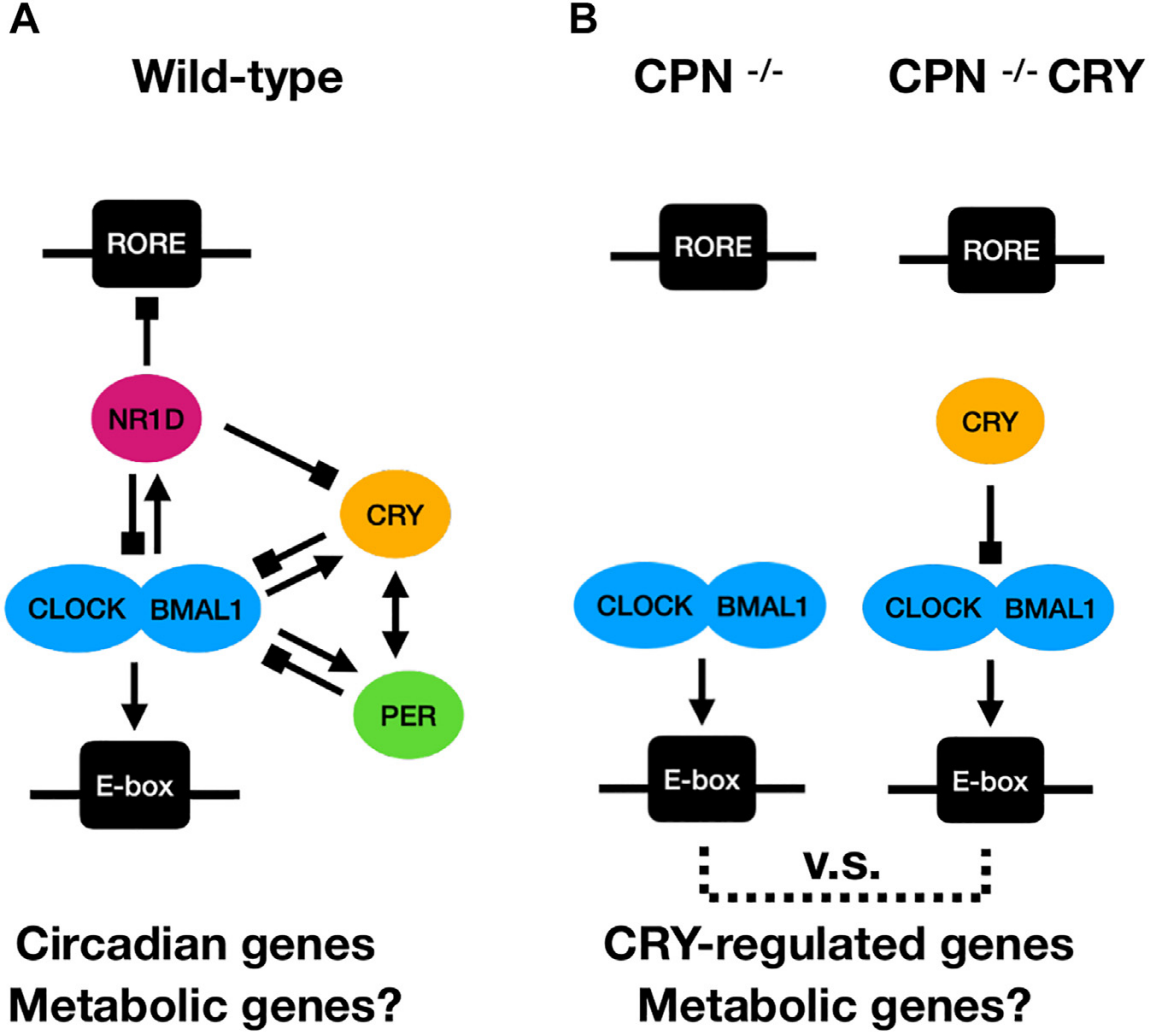
**Schematic diagrams of transcriptional regulation of circadian genes by the core clock proteins in WT, CPN**^**−/−**^**and CPN**^**−/−**^**CRY cells.***A*, core clock proteins in WT cells contain CRY, PER, and NR1D proteins. Cry, Per, and Nr1d genes have E-box elements in their promoters that can be activated by CLOCK-BMAL1 (*arrow-head*). CRY and PER proteins negatively regulate CLOCK-BMAL1 activity (*diamond-head*) but can also stabilize each other (*double arrows*). NR1D proteins repress the expression of bmal1 and Cry through promoter-based elements (*diamond-head*). The interconnected regulatory network between these core clock proteins is complex. The studies of circadian genes might also have to consider the regulation of metabolic genes. *B*, a mouse embryonic fibroblast cell line lacking CRY, PER, and NR1D (Cry1/2^−/−^; Per1/2^−/−^; Nr1d1/2^−/−^, called CPN^−/−^ in this study) has been established to facilitate the mechanism studies (26). In this study, the CPN^−/−^ CRY cell line expressing exogenous CRY proteins in the CPN^−/−^ cell line is established and is compared to the CPN^−/−^ cell line to elucidate the CRY-regulated genes and the influence of CRY on the cellular metabolism. BMAL1, brain and muscle ARNT-like 1; CLOCK, circadian locomotor output cycles kaput; CRY, cryptochrome; NR1D, nuclear receptor superfamily 1 group D.

**Figure 2:**
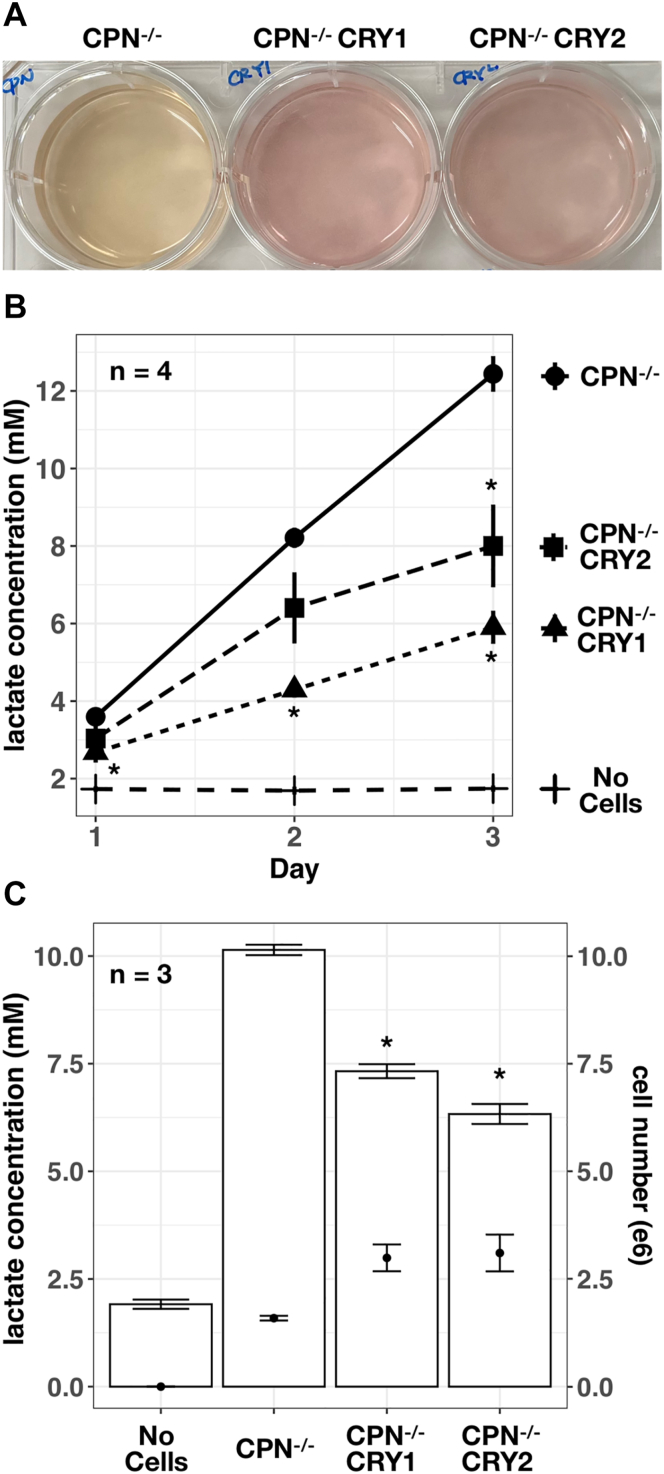
**CRY1 and CRY2 expression decreased the lactate production in mouse embryonic fibroblast cells.***A*, a representative image of the medium obtained from the CPN^−/−^ cell line and its derivatives cell lines, CPN^−/−^ CRY1 and CPN^−/−^ CRY2, is shown. The image was taken after 3 days of seeding 4 × 10^5^ cells in a 6-well plate. *B*, lactate concentration in the medium collected at 24 h (Day 1), 48 h (Day 2), and 72 h (Day 3) after seeding was determined and converted into mM using the lactate standard provided by the kit. Medium without cells (no cells) was maintained in the CO_2_ incubator as background control. Symbols: *cross* (No cells), *circle* (CPN^−/−^), *triangle* (CPN^−/−^ CRY1), and *square* (CPN^−/−^ CRY2) cells. Data expressed as mean ± standard error (n = 4). Data with statistically significant differences compared to CPN^−/−^ was marked (∗, unpaired *t* test, *p* < 0.05). *C*, bar graphs represent lactate concentration (mM, *left Y*-axis) from the culture medium from the fourth day. Cell numbers after the medium collection were determined and are displayed as *dots* within each *bar* (e6, *right Y*-axis). Data expressed as mean ± standard error (n = 3). Data with statistically significant differences to CPN^−/−^ was marked (∗, unpaired *t* test, *p* < 0.05). CRY, cryptochrome.

To study the specific function of individual proteins in the TTFL, we established a mouse embryonic fibroblast cell line lacking CRY1, CRY2, PER1, PER2, NR1D1, and NR1D2 proteins (CPN^−/−^) (26). We reconstituted CRY1 or CRY2 proteins in the CPN^−/−^ cell line by the lentiviral system to obtain CPN^−/−^ CRY1 and CPN^−/−^ CRY2 cell lines for studying CRY1 or CRY2-specific effects on the transcriptional regulation of clock-regulated genes (Fig. 1). The lentiviral reintroduction of CRY1 or CRY2 in the CPN^−/−^ cell line is suitable for the study of CRY-regulating phenomena but cannot recapitulate the transcriptional regulation of Cry1 or Cry2 gene because the exogenous expression of CRY1 or CRY2 controlled by the viral promoter, but not endogenous promoter, was not affected by the feedback regulation of the TTFL. Under the same cell culture conditions, we observed that the medium acidification of CPN^−/−^ CRY1 and CPN^−/−^ CRY2 cell lines was slower than that of the parental CPN^−/−^ cell line (Fig. 2*A*). Because lactate is the major end product in cell metabolism that caused the medium acidification in cell culture (33), we analyzed the lactate concentration in the medium of CPN^−/−^, CPN^−/−^ CRY1 and CPN^−/−^ CRY2 cell lines. Culture media were collected at the time point of 24 h (day 1), 48 h (day 2) and 72 h (day 3) after seeding the same number of cells in the plates. We found that lactate accumulation in the culture medium of the CPN^−/−^ cell line was faster than in the culture medium of CPN^−/−^ CRY1 and CPN^−/−^ CRY2 cell lines (Fig. 2*B*). Statistically significant differences between CPN^−/−^ CRY1, CPN^−/−^ CRY2 and CPN^−/−^ could be determined after 24 and 72 h, respectively. To understand whether the difference in lactate production is due to the cell numbers affected by the cell growth rate, we refreshed the medium 72 h after seeding and analyzed the lactate concentration accumulated from 72 to 96 h (the fourth day). After collecting the medium for lactate determination, we harvested the cells and counted the cell number using an automatic hemocytometer (Fig. 2*C*). We found that lactate concentration is higher in the culture medium of CPN^−/−^ cells (mean: 10.14 mM) than in the culture medium of CPN^−/−^ CRY1 (mean: 7.33 mM) and CPN^−/−^ CRY2 cells (mean: 6.33 mM). However, the cell number of all three cell lines was compatible. These data suggested that CRY1 and CRY2 proteins can decrease lactate production in mouse embryonic fibroblast cells without PER or NR1D proteins.

### CRY protein inhibited the expression of Pdk1 and Ldha in regulating lactate production

To understand the mechanism of how CRY1 and CRY2 affect lactate production, we first focused on the enzymes involved in the conversion of pyruvate into either lactate or acetyl-CoA. From the Kyoto Encyclopedia of Genes and Genomes (KEGG) database, we selected: (1) enzymes that catalyzed the conversion between lactate and pyruvate (EC 1.1.1.27), LDH, including LDHA, LDHB, LDHC, LDHAL6A, and LDHAL6B; (2) enzymes that catalyzed the conversion from pyruvate to acetyl-CoA (EC 1.2.4.1, EC 1.8.1.4, and EC 2.3.1.12), PDH complex, including PDHA1, PDHA2, PDHB, dihydrolipoyl dehydrogenase, and DLAT and (3) enzymes that regulate PDH activity by phosphorylation (EC 2.7.11.2), PDK, including PDK1, PDK2, PDK3, and PDK4.

**Figure 3:**
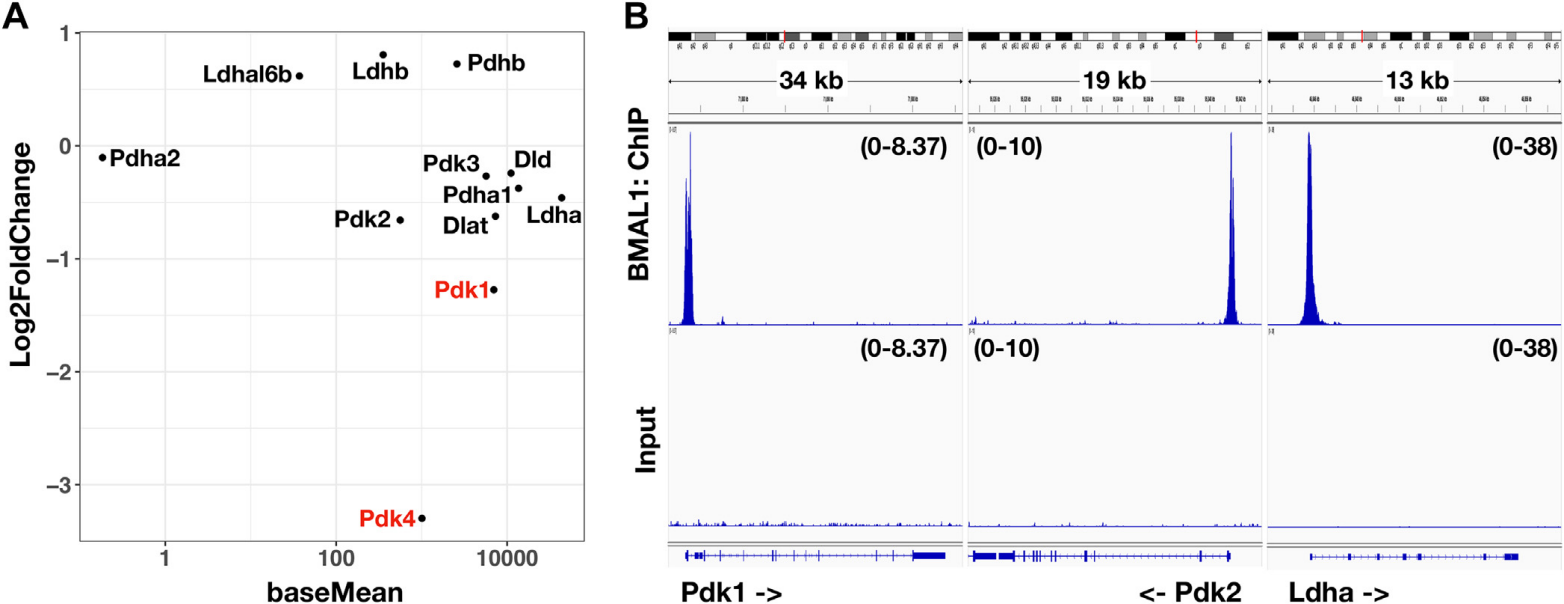
**RNA-seq and BMAL1-ChIP-seq data of CPN**^**−/−**^**and PN**^**−/−**^**cells on selected metabolic genes.***A*, relative levels of selected metabolic genes between cells expressing endogenous CRY proteins but not PER and NR1D proteins (PN^−/−^) and the CPN^−/−^ cell line from published RNA-seq data (GSE157946) were plotted. The *X*-axis (baseMean) represents the relative levels between different genes. The *Y*-axis (log2FoldChange) represents the relative levels of mRNAs in PN^−/−^ cells *versus* CPN^−/−^ cells. Pdk1 and Pdk4 showed statistically significant differences between these cell lines (*red*). *B*, screenshots of BMAL1-ChIP-seq data (GSE93318) on the genome browsers at the regions of Pdk1, Pdk2, and Ldha genes. Sequencing data from unenriched fragmented DNA before IP (input) are shown for comparison. Signal scales are shown in the parenthesis. BMAL1, brain and muscle ARNT-like 1; ChIP, chromatin immunoprecipitation; CRY, cryptochrome; IP, immunoprecipitation; Ldha, lactate dehydrogenase A; NR1D, nuclear receptor superfamily 1 group D; Pdk, pyruvate dehydrogenase kinase.

We analyzed our published RNA-Seq data of CPN^−/−^ cells as well as another mouse embryonic fibroblast cell line with endogenous CRY proteins but lacking PER and NR1D proteins (PN^−/−^) (GSE157946) (26). Two genes selected from the KEGG database could not be analyzed, because there is no Ldhal6a gene in the annotated mouse genome and Ldhc mRNA was not detected in this dataset. From this dataset, Pdk1 and Pdk4 mRNA were significantly lower in the PN^−/−^ cell line than in the CPN^−/−^ cell line (Fig. 3*A*). We also analyzed the BMAL1 occupation on the promoter of all candidate genes using published BMAL1-chromatin immunoprecipitation (ChIP)-Seq data from PN^−/−^ cells expressing PER2 fused with the ligand binding domain of estrogen receptor (GSE93318) (8). BMAL1 did bind to the promoter region of Pdk1, Pdk2, and Ldha genes (Fig. 3*B*). According to the RNA-Seq results comparing cell lines with endogenous CRY (PN^−/−^) and without endogenous CRY (CPN^−/−^) and the BMAL1 occupation on the promoter in the PN^−/−^ cell line, Pdk1 is the primary target for further analysis. Although there was no statistically significant difference in the expression of Ldha between CPN^−/−^ and PN^−/−^ cell lines, Ldha expression in PN^−/−^ cell line was around 70% of that in CPN^−/−^ cell line. Furthermore, the BMAL1 binding signal of the Ldha promoter was around 4-fold stronger than the signal of the Pdk1 promoter. Thus, Ldha is another target we selected for studying CRY-mediated inhibition of lactate production.

**Figure 4:**
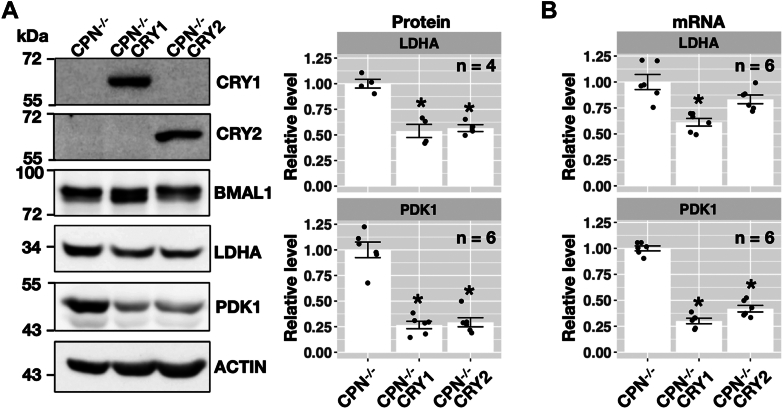
**Analysis of protein and mRNA levels of LDHA and PDK1 in the CPN**^**−/−**^**, CPN**^**−/−**^**CRY1 and CPN**^**−/−**^**CRY2 cells.***A*, representative Western blot results of LDHA and PDK1 are shown in the *left panel*. ACTIN was used as the loading control. The relative phosphorylation level of BMAL1 changed when CRY1 or CRY2 was expressed (upper band *versus* lower band). Relative LDHA and PDK1 levels were quantified and expressed as mean ± standard error. *Dots* indicate individual data from independent experiments. Data with statistically significant differences to CPN^−/−^ cells was marked (∗, unpaired *t* test, *p* < 0.05). *B*, relative LDHA and PDK1 mRNA levels were quantified by real-time RT-PCR and expressed as mean ± standard error. Data with statistical differences to CPN^−/−^ cells was marked (∗, unpaired *t* test, *p* < 0.05). BMAL1, brain and muscle ARNT-like 1; CRY, cryptochrome; Ldha, lactate dehydrogenase A; Pdk, pyruvate dehydrogenase kinase; RT-PCR, reverse transcription PCR.

We hypothesized that CRY proteins could inhibit lactate production by not only decreasing the LDHA protein level but also enhancing PDH activity by reducing PDK1-mediated phosphorylation. Compared to CPN^−/−^ cell line, the expression of CRY1 protein in CPN^−/−^ CRY1 cell line or expression of CRY2 protein in CPN^−/−^ CRY2 cell line decreased LDHA and PDK1 protein levels (Fig. 4*A*). The RNA level analysis showed that LDHA mRNA in CPN^−/−^ CRY1 cell line was lower than in CPN^−/−^ cell line. Expression of CRY2 in CPN^−/−^ CRY2 cell line did not statistically significantly affect the LDHA mRNA but affected the LDHA protein. Furthermore, PDK1 mRNA levels were lower in both CPN^−/−^ CRY1 and CPN^−/−^ CRY2 cell lines compared to the mRNA level in CPN^−/−^ cell line (Fig. 4*B*). These data suggested that CRY proteins inhibited the transcription of Ldha and Pdk1 and decreased LDHA and PDK1 proteins (increasing PDH activity) inhibited lactate production.

### CRY protein inhibited the expression of Pdk1 and Ldha by binding to the E-box of their promoters

**Figure 5:**
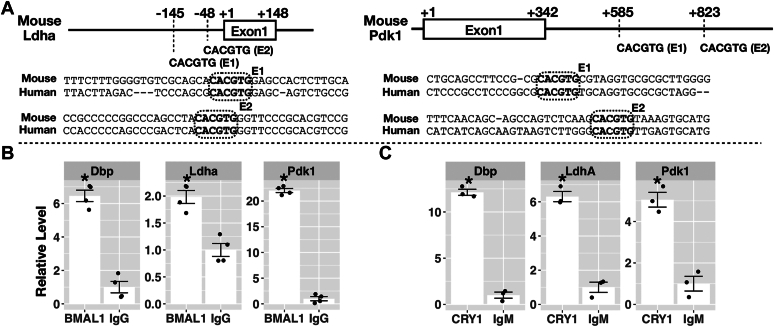
**Chromatin immunoprecipitation (ChIP) analysis of BMAL1 and CRY1 on Pdk1 and Ldha promoter.***A*, relative positions of canonical E-box sequences (CACGTG) around the transcription start (+1) of mouse Ldha and Pdk1 genes are shown. Two E-boxes (E1 and E2) were identified for both genes. Neighboring sequences around the E-boxes are shown and were compared to the human homologous genes. *B*, relative levels of DNA fragments pull-down by the anti-BMAL antibody to DNA fragments pull-down by the control IgG antibody from CPN^−/−^ cells measured by real-time PCR analysis. Specific primer sets closed to the E-boxes of Dbp (positive control), Ldha and Pdk1 were used to quantify pull-down DNA fragments. Data were expressed as mean ± standard error. *Dots* represent individual data from four independent experiments. Data with statistically significant differences to the IgG control were marked (∗, unpaired *t* test, *p* < 0.05). *C*, relative levels of DNA fragments pull-down by anti-CRY1 antibody to DNA fragments pull-down by the control IgM antibody from CPN^−/−^ CRY1 cells measured by real-time PCR analysis. Primer sets used in CRY1-ChIP were the same as BMAL1-ChIP. Data were expressed as mean ± standard error. Dots represent individual data from three independent experiments. Data with statistically significant differences to the IgG control were marked (∗, unpaired *t* test, *p* < 0.05). BMAL1, brain and muscle ARNT-like 1; ChIP, chromatin immunoprecipitation; CRY, cryptochrome; Dbp, D site albumin promoter binding protein; Ig, immunoglobulin; Ldha, lactate dehydrogenase A; Pdk, pyruvate dehydrogenase kinase.

To understand whether CRY inhibited the transcription of Pdk1 and Ldha through CLOCK-BMAL1 binding to the E-box, we analyzed their promoter sequences and performed ChIP using BMAL1 and CRY1 antibodies. Previous BMAL1-ChIP-seq data from PN^−/−^ cells (GSE93318) showed signals within −452 ∼ +256 region (708 bp) of the Ldha gene and −35 ∼ +1051 region (1086 bp) of the Pdk1 gene. Both regions contain two canonical E-box sequences (CACGTG) also conserved in the human LdhA and Pdk1 genes (Fig. 5*A*). The E-boxes of Ldha are located on the −145 and −48 upstream of the first exon. The E-boxes of Pdk1 are located on the +585 and +823 within the first intron. We performed BMAL1-ChIP using CPN^−/−^ cells (Fig. 5*B*) and performed CRY1-ChIP using CPN^−/−^ CRY1 cells (Fig. 5*C*). BMAL1-ChIP and CRY1-ChIP showed significant enrichment of DNA fragments from the promoter of the D site albumin promoter binding protein (Dbp) gene, which is a known CLOCK-BMAL1-regulated gene (4). Furthermore, we showed that BMAL1 and CRY1 can bind to the promoters of Pdk1 and Ldha suggesting that the expression of these genes can be regulated by the CLOCK-BMAL1 and CRY through E-box similar to other primary clock-controlled genes, like Dbp, Per1, and Per2.

### CRY protein decreased the expression of glycolysis genes

**Figure 6:**
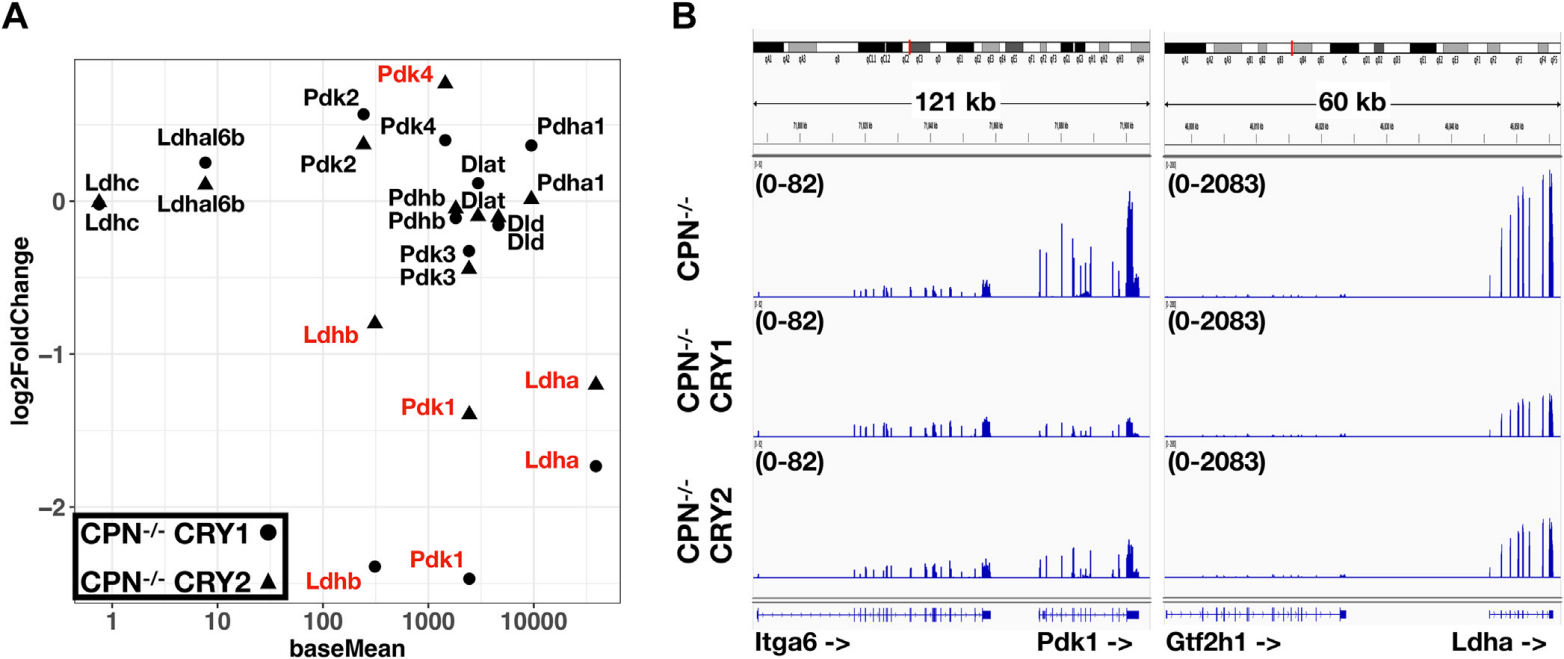
**RNA-seq data of CPN**^**−/−**^**CRY1, CPN**^**−/−**^**CRY2 and CPN**^**−/−**^**cells on selected metabolic genes.***A*, relative mRNA levels of selected metabolic genes between CPN^−/−^ CRY1 or CPN^−/−^ CRY2 *versus* CPN^−/−^ cell lines from mRNA sequencing data in this study were plotted. The *X*-axis (baseMean) represented the relative levels between different genes. The *Y*-axis (log2FoldChange) represented the relative levels of mRNAs in CPN^−/−^ CRY1 (*circle*) or CPN^−/−^ CRY2 (*triangle*) cells *versus* CPN^−/−^ cell line. Genes showed statistically significant differences in mRNA level compared to CPN^−/−^ cell line were marked (*red*). *B*, screenshots of mRNA-seq data in this study on the genome browsers at the regions of Pdk1 and Ldha genes are shown. Signal scales are shown in the *parenthesis*. CRY, cryptochrome; Ldha, lactate dehydrogenase A; Pdk, pyruvate dehydrogenase kinase.

**Figure 7:**
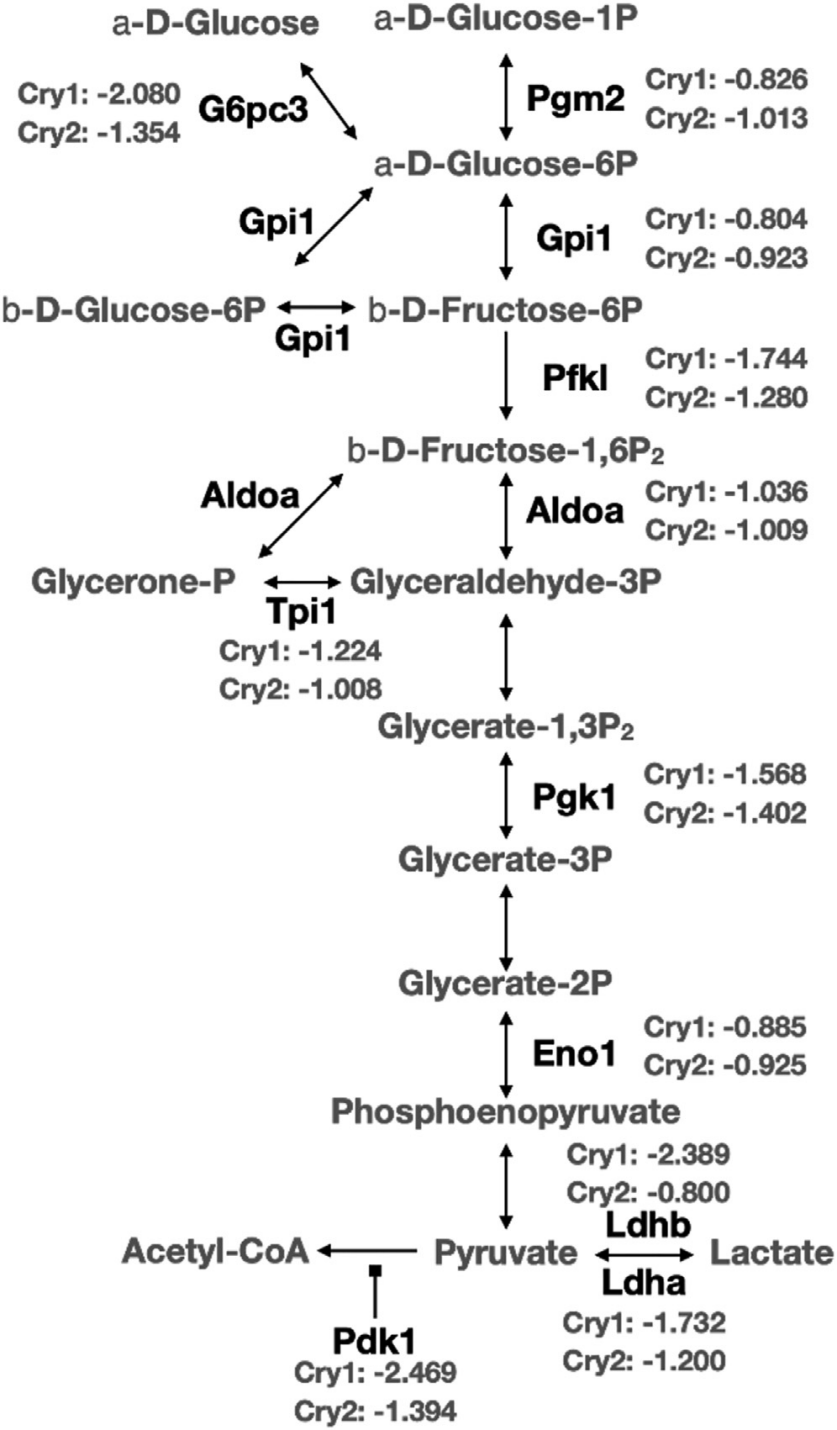
**Gene enrichment analysis of RNA-seq data from CPN**^**−/−**^**CRY1 and CPN**^**−/−**^**CRY2 cells *versus* RNA-seq data from CPN**^**−/−**^**cells.** Differentially expressed genes in the glycolysis pathway are shown. Relative expression levels of these genes in CPN^−/−^ CRY1 or CPN^−/−^ CRY2 cells *versus* CPN^−/−^ cells were expressed as log_2_ (Fold). CRY, cryptochrome.

**Supplemental Figure S1:**
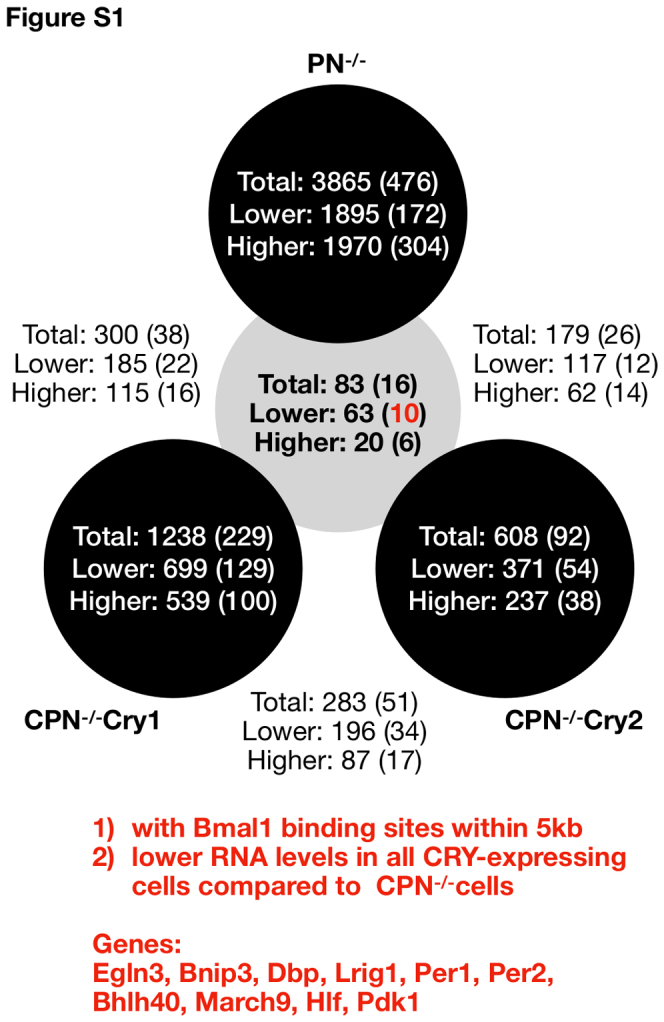


As lactate is a product of glycolysis, we wanted to analyze the effects of CRY proteins on the expression of genes involved in glycolysis. For that purpose, we performed mRNA-seq analysis of CPN^−/−^, CPN^−/−^ CRY1 and CPN^−/−^ CRY2 cell lines. There are 1238 and 608 genes affected by the expression of CRY1 or CRY2 in CPN^−/−^ cell line, respectively. We also compared these CRY-affected genes with genes differentially expressed between PN^−/−^ and CPN^−/−^ cell lines and with putative BMAL1-regulated genes according to the previous BMAL1-ChIP-seq data (Fig. S1 and Table S1). About 12 to 18% of CRY-affected genes overlapped with the BMAL1 binding sites within 5 kb. Although CRY proteins were known as transcriptional repressors, the number of upregulated genes in CRY-expressing cells was similar to the number of downregulated genes. We checked genes involved in lactate production (as previously mentioned) from the mRNA-seq data (Fig. 6). PDK1 and LDHA mRNA were statistically significantly lower in CPN^−/−^ CRY1 and CPN^−/−^ CRY2 cell lines than in CPN^−/−^ cell line, consistent with the real-time reverse transcription PCR (real-time RT-PCR) analysis (Fig. 4*B*). LDHB mRNA was also statistically significantly lower in CPN^−/−^ CRY1 and CPN^−/−^ CRY2 cell lines, although there was no difference between CPN^−/−^ cell line and PN^−/−^ cell line, that expressed endogenous CRY1 and CRY2 proteins (Fig. 3). Previous BMAL1-ChIP cannot detect the binding of BMAL1 the promoter of Ldhb. The effects of CRY proteins on Pdk4 expression were complex. PDK4 mRNA was higher in the CPN^−/−^ CRY2 but not in the CPN^−/−^ CRY1 compared to the CPN^−/−^ suggesting that CRY2, but not CRY1, may enhance the expression. However, PN^−/−^ cell line expressing both CRY proteins inhibited the expression of Pdk4. Furthermore, we analyzed the pathways affected by CRY1 and CRY2 by gene set enrichment analysis (GSEA) of hallmark pathways (Table S2) and gene ontology enrichment analysis of KEGG pathways (Table S3). Glycolysis was one of the pathways affected by CRY1 and CRY2 in both analyses. Genes involved in different steps of glycolysis, including Pgm2, G6pc3, Gpi1, Pfkl, Aldoa, Tpi1, Pgk1, and Eno1, showed lower expression levels in the CPN^−/−^ CRY1 and CPN^−/−^ CRY2 cell lines compared to CPN^−/−^ cell line (Fig. 7). Our data suggested that besides affecting the pyruvate conversion to lactate or acetyl-CoA through regulating Ldha and Pdk1 expression, CRY inhibits glycolysis by repressing the expression of multiple genes in glycolysis.

### CPN^−/−^ CRY1 cell line use less glucose but are more sensitive to glucose starvation compared to CPN^−/−^ cell line

**Figure 8:**
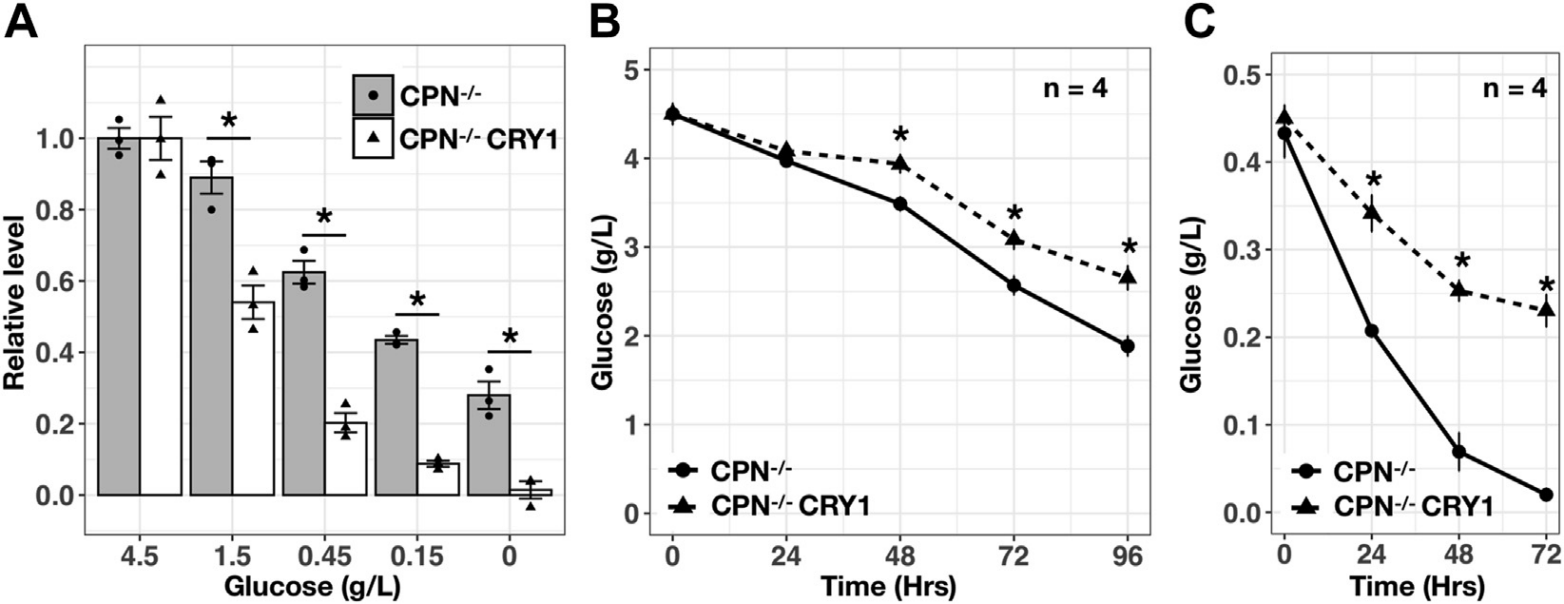
**Cell growth and glucose usage of CPN**^**−/−**^**and CPN**^**−/−**^**CRY1 cells at different glucose concentrations.***A*, cell numbers (indicated by Resazurin assay) of CPN^−/−^ and CPN^−/−^ CRY1 cell lines cultured in the medium with varying glucose concentrations compared to the cell numbers of these cell lines cultured in the high-glucose (4.5 g/l) medium. Results from four independent experiments were expressed as mean ± standard error. Individual data are shown as *dots*. Data with statistically significant differences to CPN^−/−^ cells was marked (∗, unpaired *t* test, *p* < 0.05). *B*, glucose concentrations in the medium collected 24, 48, 72, and 96 h after seeding were determined by colorimetric glucose assay and converted into g/l using culture medium without cells as standard (4.5 g/l). Data expressed as mean ± standard error (n = 4). Data with statistically significant differences to CPN^−/−^ cells was marked (∗, unpaired *t* test, *p* < 0.05). *C*, experiments were performed as in (*B*), but the glucose concentration in the original culture medium was 0.45 g/l. CRY, cryptochrome.

We observed that lactate production and glycolysis could be inhibited by CRY proteins through transcriptional regulation. We assumed that cells utilizing more glycolysis for ATP synthesis spend more glucose because of the low yield of ATP synthesis per glucose through glycolysis. This could mean that the changed glycolysis levels may affect the response of cells when glucose concentration decreases. We used resazurin assay to indirectly indicate the cell numbers of CPN^−/−^ and CPN^−/−^ CRY1 cell lines grown in media with different concentrations of glucose (Fig. 8*A*). Both cell lines showed decreased cell numbers when glucose concentration decreased. CPN^−/−^ CRY1 cell line was more sensitive to glucose starvation than CPN^−/−^ cell line. We next analyzed the glucose usage of CPN^−/−^ and CPN^−/−^ CRY1 cell lines by determining the remaining glucose concentration in the culture medium on different days. Glucose concentration decreased faster in the culture medium of CPN^−/−^ cell line than in the medium of CPN^−/−^ CRY1 cell line (Fig. 8*B*) suggesting that CPN^−/−^ utilized more glucose than CPN^−/−^ CRY1. We performed the same glucose usage analysis in the medium with low glucose concentration (0.45 g/l, 10% of regular medium). Similarly, CPN^−/−^ consumed more glucose than CPN^−/−^ CRY1 until the glucose was almost exhausted after 72 h (Fig. 8*C*).

### CRY1 inhibits Pdk1 expression, PDH phosphorylation, glucose expenditure, and growth in MDA-MB-231 breast cancer cell line

Previous studies have indicated that circadian disruption is a known risk factor for breast cancer (34). Aerobic glycolysis, or the Warburg effect, is a common property of cancer cells (35). We attempted to test whether CRY1 could suppress the growth of cancer cells by decreasing glycolysis. For that purpose, we chose the MDA-MB-231 cell line (MB231), a representative cell line of triple-negative breast cancer, which lacks expression of estrogen receptor, progesterone receptor, and human epidermal growth factor receptor 2 (36). In addition, it has been reported that PDK1 level was positively correlated with the glycolysis level and the stemness in the MDA-MB-231 cell line (32).

**Figure 9:**
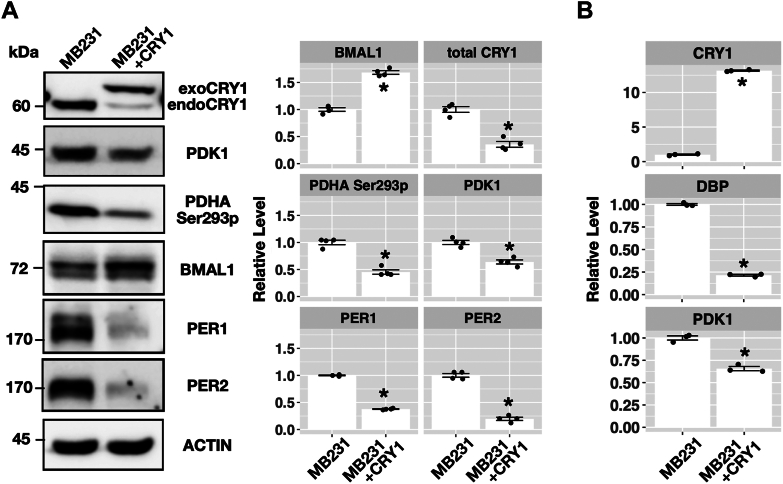
**Analysis of the influence of CRY1 on the expression of PDK1 in the MDA-MB231 cells.***A*, representative Western blot results of PDK1, PDH phosphorylation (PDHA Ser293p), BMAL1, PER1, PER2, and CRY1 in the MDA-MB-231 (MB231) cells and its derivative expressing exogenous CRY1 protein (MB231+CRY1). ACTIN served as the loading control. Relative protein levels were quantified and described as mean ± standard error. Data with statistically significant differences to MB231 cells was marked (∗, unpaired *t* test, *p* < 0.05). The decreased PER1 and PER2 proteins supported the activity of exogenous CRY1 protein. *B*, relative CRY1, PDK1, and DBP mRNA levels were quantified by real-time RT-PCR and expressed as mean ± standard error. Data with statistically significant differences to MB231 cells was marked (∗, unpaired *t* test, *p* < 0.05). Decreased DBP mRNA levels in MB231+CRY1 cells suggested the activity of exogenous CRY1 protein. BMAL1, brain and muscle ARNT-like 1; CRY, cryptochrome; DBP, D site albumin promoter binding protein; PDH, pyruvate dehydrogenase; Pdk, pyruvate dehydrogenase kinase; RT-PCR, reverse transcription PCR.

We established a derivative MDA-MB-231 cell line that stably expresses exogenous human CRY1 (MB231+CRY1) as demonstrated by Western blot analysis (Fig. 9*A*) (slower migrating than the endogenous CRY1 due to the presence of molecular tags). The amount of total CRY1 protein level in MB231+CRY1 was less than the parental MB231 cell line. To validate the function of the exogenous CRY1 protein, we assessed the protein levels of core clock proteins including BMAL1, PER1, and PER2. Protein levels of PER1 and PER2 in MB231+CRY1 cell line were lower than in the MB231 cell line possibly due to the transcriptional repression of Per1 and Per2 by CRY1. Higher BMAL1 protein levels in the MB231+CRY1 cell line than in the MB231 cell line were expected because of the secondary effect of CRY1 on the Bmal1 expression by decreasing the transcription of Nr1d genes, and thus NR1D proteins. Decreased DBP mRNA, a known CLOCK-BMAL1 target gene, in MB231+CRY1 cell line (Fig. 9*B*) indicated the decreased CLOCK-BMAL1 activity by expressing CRY1. Notably, mRNA levels of CRY1, including endogenous and exogenous CRY1 mRNA, in MB231+CRY1 were more than 10-fold higher than in the parental cell line. Although endogenous Cry1 expression was repressed by the exogenous CRY1 protein (lower band in CRY1 Western blot data, Fig. 9*A*), posttranscriptional mechanisms caused the decreased total CRY1 proteins. Decreased PER protein levels in MB231+CRY1 cell line, leading to decreased CRY-PER interaction, may cause the instability of CRY1 (37).

**Figure 10:**
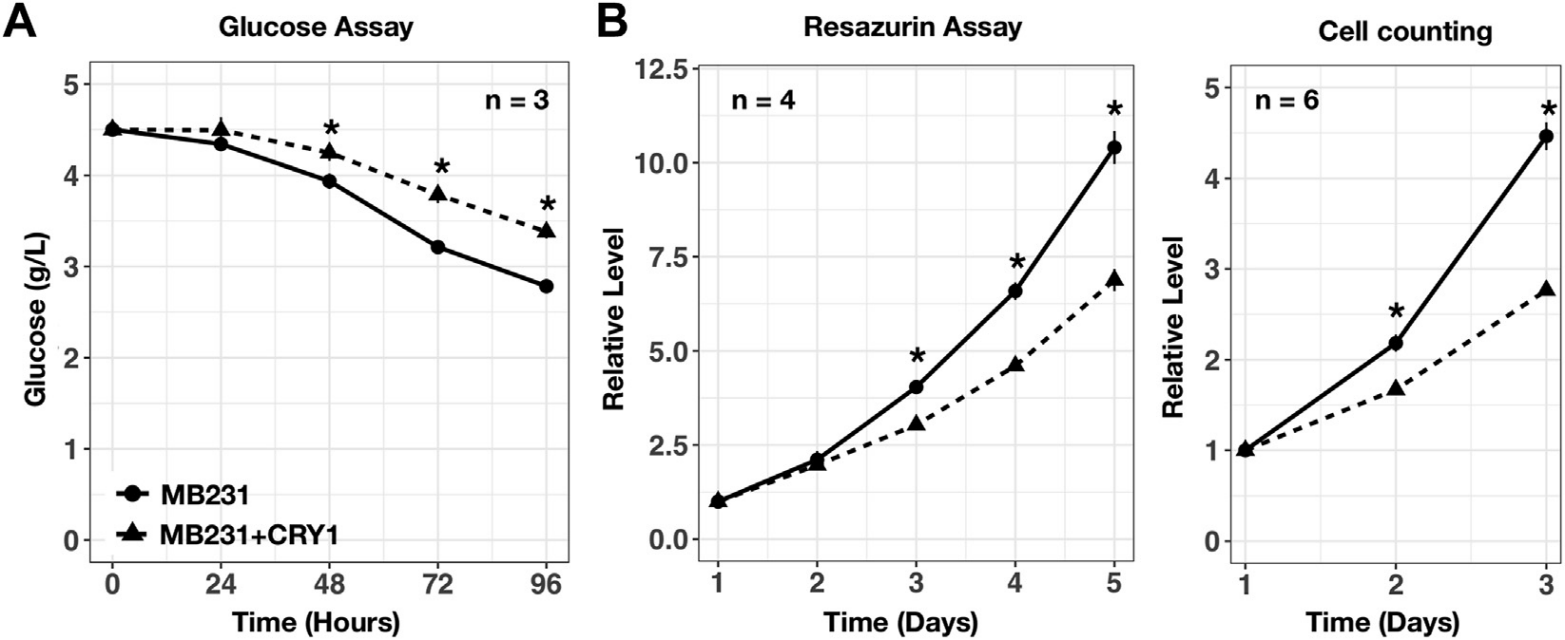
**Glucose usage and cell growth in MB231 and MB231+CRY1 cells.***A*, glucose concentrations in the medium collected 24, 48, 72, and 96 h after seeding were determined by colorimetric assay and converted into g/l using culture medium without cells as standard (4.5 g/l). Data expressed as mean ± standard error (n = 3). Data with statistically significant differences to MB231 cells was marked (∗, unpaired *t* test, *p* < 0.05). *B*, cell growth of MB-231 and MB231+CRY1 cells was estimated by Resazurin assay (*left panel*) and cell counting (*right panel*). Results were expressed as mean ± standard error. Data with statistically significant differences to MB231 cells was marked (∗, unpaired *t* test, *p* < 0.05). CRY, cryptochrome.

As in CPN^−/−^ CRY1 cell line, constitutively expressed CRY1 in MB231+CRY1 cell line led to decreased mRNA and protein levels of PDK1 (Fig. 9, *A* and *B*). It was previously shown that PDH subunit E1A serine 293 phosphorylation (PDHA Ser293p) contributed to the inactivation of PDH, a process that could be catalyzed by PDK1 (38). In MB231+CRY1 cell line, phosphorylation of PDHA Ser293 was also lower than in the MB231 cell line (Fig. 9*A*) suggesting that decreased PDK1 expression by CRY1 increases the PDH activity by decreasing the phosphorylation. We also analyzed the glucose expenditure of MB231+CRY1 compared to its parental cell line. As in the CPN^−/−^ CRY1 cell line, expressing CRY1 in MB231+CRY1 cell line decreased the rate of glucose usage (Fig. 10*A*). The decreased glucose expenditure was also associated with the lower cell growth rate determined by resazurin assay or direct cell counting (Fig. 10*B*).

**Figure 11:**
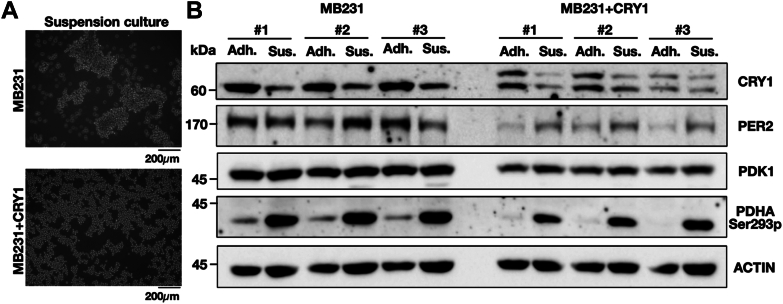
**Analysis of CRY1, PER2, and PDK1 protein levels and PDH phosphorylation of MB231 and MB231+CRY1 cells in adhesion or suspension culture.***A*, representative images of MB231 and MB231+CRY1 cells grown in suspension are shown. The scale bar in the images corresponds to a length of 200 mm. *B*, Western blot results of PDK1, PDH phosphorylation (PDHA Ser293p), PER2, and CRY1 in the MB231 cells and MB231+CRY1 cells grown in adhesion (Adh.) or suspension (Sus.) are shown. ACTIN served as the loading control. Three independent cell sets (#1, #2 and #3) were collected and analyzed. CRY, cryptochrome; PDH, pyruvate dehydrogenase; Pdk, pyruvate dehydrogenase kinase.

Tumor cells growing in a three-dimensional environment can better mimic the architecture and cell-cell interaction *in vivo* than growing on a two-dimensional plate. This could be tested using ultra-low attachment plates. We found that MB231 cells formed bigger cell clusters than MB231+CRY1 cells under the same culture condition (Fig. 11*A*). We also analyzed whether the CRY1-induced decrease of PDK1 could be observed in the MB231+CRY1 cell line that grew in suspension conditions. Using Western blot analysis, we found less PDK1 protein in MB231+CRY1 cell line than in MB231 cell line in adhesion and suspension cultures. In addition, both cell lines expressed less CRY1 and more PDHA Ser293 phosphorylation when grown in suspension compared to growth in adhesion conditions. In MB231+CRY1 cell line, decreased CRY1 protein in suspension culture was associated with the higher expression of PDK1, PER2, and PDHA Ser293 phosphorylation (Fig. 11*B*). We also performed experiments using another breast cancer cell line, MCF-7, to analyze the correlation between CRY1 and PDK1 proteins. Like MB231+CRY1 cell line, in MCF-7 cell line, decreased CRY1 protein in suspension culture was associated with increased PDK1 protein and PDHA Ser293 phosphorylation (Fig. S2*A*). In addition, we also observed that PDK1 and CRY1 proteins in MCF-7 cell line responded reversely after serum synchronization (Fig. S2*B*). These data suggested that CRY1 protein can affect the expression of PDK1 and, thus, the change in cell growth. The data also suggested that cells could adapt to environmental changes by modulating the PDK1 through CRY1.

## Discussion

### CRY proteins shifted glucose metabolism from glycolysis to the TCA cycle

The circadian clock generates the daily rhythmicity of many biological processes. Glucose is essential for most cells to provide energy or intermediates for other biomolecules through different metabolic pathways. Circadian control of glucose homeostasis *in vivo* has previously been discussed (39, 40, 41). The mechanisms include central clock-controlled behaviors, feeding and sleep, and metabolic rhythmicity through peripheral clocks in the liver, pancreas, adipose tissue, and muscles. However, whether circadian control of glucose metabolism happens at the cellular level and if so, its understanding is unknown. Our study here provides additional data showing evidence that CRY proteins (CRY1 and CRY2) could inhibit the expression of Pdk1, Ldha, and other genes involved in glycolytic pathways suggesting that the core clock mechanism contributes to the glucose metabolic switch in mammalian cells.

**Figure 12:**
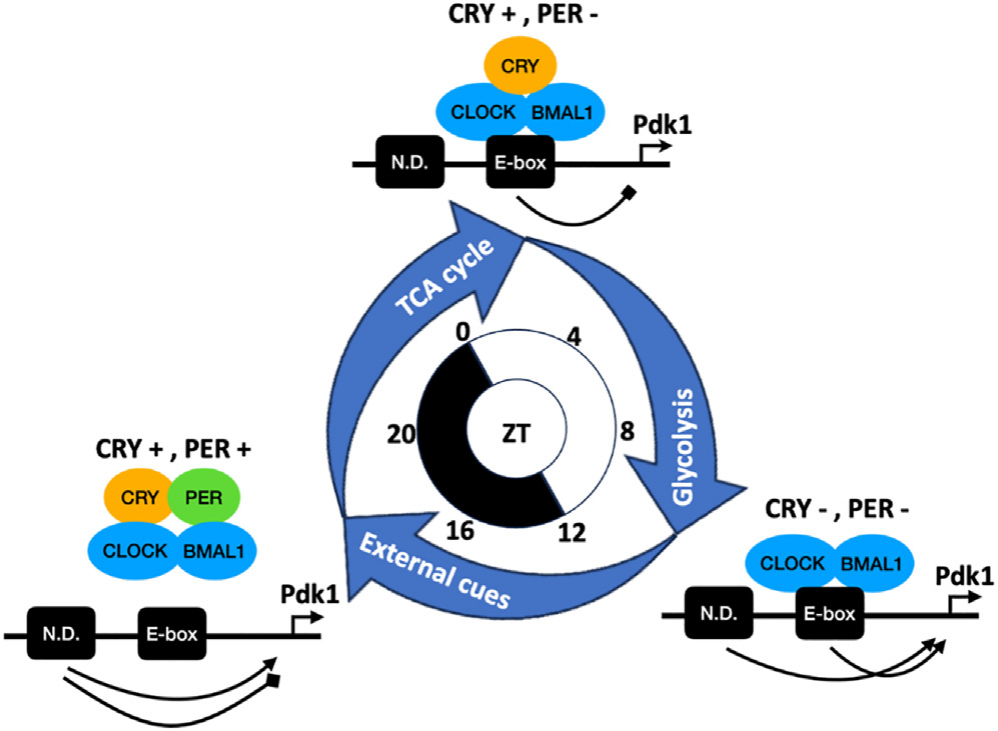
**The model of regulations of glucose metabolic pathways by the circadian clock's transcription-translation feedback loop (TTFL).** This model describes how CLOCK-BMAL1 dimer, CRY, and PER can regulate the expression of metabolic regulator Pdk1 and modulate the glucose metabolic pathways at different times of the day. Protein complexes associated with specific Zeitgeber time (ZT) in the 12-h light/12-h dark condition are proposed according to the abundance of CRY and PER in the cell nuclei of mouse liver. When mice become physically active after light-off (ZT 12), Pdk1 expression can be either activated (*arrow-head*) or repressed (*diamond-head*) through not-yet-defined (N. D.) elements in its promoter responding to external cues. CLOCK-BMAL1 gradually decreases the control of Pdk1 expression because the accumulation of the CRY-PER complex displaces its binding on the E-box element (ZT 16–20). Because expression of the Cry gene, but not the Per gene, is not CLOCK-BMAL1-dependent, accumulated CRY protein represses Pdk1 expression, shifting the metabolic pathways to TCA-dominated. After CRY protein reaches the peak level (ZT 0–4), the feedback regulation in the TTFL (through CRY-CLOCK-BMAL1) will decrease it again. CRY-mediated repression of Pdk1 is released and redirects the metabolic pathways to glycolysis-dominated. When the CRY protein level drops to the trough (ZT 8–12), CLOCK-BMAL1 activates the transcription of Per, and a new regulation cycle starts. BMAL1, brain and muscle ARNT-like 1; CLOCK, circadian locomotor output cycles kaput; Pdk, pyruvate dehydrogenase kinase.

Here, we proposed a time-dependent regulatory model of cellular glucose metabolism based on the TTFL model, since CRY can inhibit glycolysis combined with the relative quantity and the distinct regulatory mechanism of CRY and PER proteins (Fig. 12). Mice are physically active after light-off (Zeitgeber time, ZT 12) and inactive after light-on (ZT 0) in a regular 12-h light and 12-h dark environment (42). CLOCK-BMAL1 shows maximal activity for transcriptional activation of clock genes hours before light-off (ZT 8–ZT 12). Then, PER protein accumulates and reaches the maximal level around ZT 16 to ZT 20. CRY protein accumulates with some delay due to the transcriptional repression by NR1D proteins. CRY-PER complex displaces CLOCK-BMAL1 from promoters of target genes and increases the sensitivity of Pdk1 expression to noncircadian regulators. Because PER protein reduces the level of its own, but not CRY, more CRY protein accumulates relative to PER at the late physically active phase and early inactive phase (ZT 0–ZT 4). CRY protein downregulates the expression of Pdk1 and shifts the glucose metabolism toward the TCA cycle. CRY protein reduces the levels of PER, CRY, and NR1D, recovering the activity of CLOCK-BMAL1, and the glucose metabolism shifts again from the TCA cycle toward glycolysis. The cycle restarts when the CRY level reaches the trough, and the CLOCK-BMAL1 activity reaches the peak (ZT 8–ZT 12). We hypothesize that cells can control glucose metabolism in clock-dependent and clock-independent ways. During the physically active phase, clock-independent regulation is the predominant one responding to divergent external cues. During the inactive phase, external stimuli were relatively homeostatic. The clock-dependent regulation generates the time-specific balance of glucose utilization between glycolysis and the TCA cycle.

### Rhythmicity of Pdk1 expression in the mammalian circadian system

In this study, we showed that CRY proteins could repress the expression of Pdk1 in a noncircadian cell system. Because CRY1 and CRY2 were core TTFL proteins with oscillated protein levels, and Pdk1 expression is regulated by the CRY protein, we expected that Pdk1 might be a circadian gene having rhythmic expression. We searched the CIRCA database (43) to understand whether the rhythmic expression of Pdk1 could be observed in the mammalian circadian system. From the CIRCA database, rhythmic mRNA levels could be detected in the liver and aorta (JTK Q-value < 0.05) of mice and the artery, colon, and epidermis (false discovery rate < 0.05) of humans. We also searched another data set of time-series RNA sequencing data from different mouse tissues (GSE54651) (1). Pdk1 expression showed rhythmicity in the brain stem, kidney and liver by analyzing this dataset using the meta2D method of MetaCycle (44). This information suggested that Pdk1 expression could be regulated by the circadian clock at least in some tissues. However, we cannot know whether the regulation by the core clock is sufficient to generate the rhythmicity of Pdk1 *in vivo*. In another way, divergent environmental stimulations in the physically active phase increased the difficulty of identifying the rhythmicity of Pdk1. Further studies focusing on the time between the early and late rest phase with different CRY levels but under constant environmental conditions would be informative to understand the influence of CRY on the Pdk1 expression and the glucose metabolism *in vivo*.

### Cross talk of the clock and oxygen in the regulation of glycolysis

Oxygen is a key factor in regulating glycolysis. When the oxygen level is insufficient, the hypoxia signaling pathways will become activated and trigger the anaerobic glycolysis to provide sufficient ATP (27). HIF1α is essential for this metabolic shift by activating the transcription of genes for glycolysis, including Ldha (45), and Pdk1 (46). In contrast, in this study we found that CRY proteins repress the transcription of Ldha and Pdk1. Although the mechanism of how HIF1α and the core clock coregulate Pdk1 or glycolysis is unclear, the reciprocal regulation of the circadian clock and hypoxia has been observed. BMAL1 may regulate HIF1α downstream genes by regulating HIF1α transcription because BMAL1 can bind to the promoter of HIF1α and activate its transcription (28). In addition, BMAL1 cistrome and HIF1α cistrome partially overlap, and the expression of 10 to 20 percent hypoxia-responsive genes oscillated with daylight pattern, suggesting the cross talk between the circadian clock and the hypoxia responses (28, 31). Besides interacting with CLOCK-BMAL1, CRY proteins have been reported to interact with HIF1α providing an additional mechanism of how the circadian clock affects hypoxic response (21, 47). In this study, we found that CRY protein could downregulate the expression and Pdk1, Ldha, and glycolytic genes under normal oxygen concentration. In this condition, we did not observe the difference in HIF1α mRNA from our mRNA-seq data between the CPN^−/−^ and CPN^−/−^ CRY1 cell lines. Thus, we thought the repression of glycolytic genes by the CRY protein in this study was not HIF1-dependent. These genes can be regulated by both the circadian clock through the TTFL and the oxygen concentration through the HIF1α pathway. Further studies are needed to understand whether the HIF1α could activate CRY-mediated repression of Pdk1 and whether CRY could repress HIF1-induced Pdk1 expression to dissect the mechanism of glycolysis regulation.

### Role of CRY in the Warburg effect of cancer

Many tumor cells have a characteristic preference to convert glucose to lactate through glycolysis even under normal oxygen concentration, called aerobic glycolysis or the Warburg effect (35). Researchers endeavor to target the Warburg effect for cancer treatment by remodeling the metabolism of the cancer cells (48, 49). Aerobic glycolysis in cancer cells involves changes in multiple regulatory networks and the transcriptional activation of key enzymes regulating glycolysis, including Ldha and Pdk1 (50). The role of the core clock mechanism, the TTFL, in aerobic glycolysis is unclear. Rhythmicity of Bmal1 and Per2 promoters in MDA-MB-231 breast cancer cells after serum synchronization could not be observed (51). The inability of synchronization may reflect the dysfunction of the TTFL in this cancer cell line. The CPN^−/−^ cells showed similar phenotypes as aerobic glycolysis including more lactate production and the transcriptional activation of genes for glycolysis. However, loss of CRY expression may not be the cause of increased PDK1 expression in the MDA-MB-231 cells because exogenous expression of CRY1 did not cause the increase of total CRY1 protein level but inhibited the expression of PDK1 probably by decreasing the PER1 and PER2 protein to enhance the CRY activity (Fig. 9). Although the oncogenic function of CRY1 has been proposed by regulating DNA repair (52), the possibility that CRY1 could repress aerobic glycolysis observed in cancer cells could not be excluded. Similarly, we do not claim that CRY1 is a tumor suppressor gene because glycolysis is one of the cellular functions controlled by the circadian clock. Different functions controlled by the circadian clock could contribute differently to cancer cells. Furthermore, the rhythmicity and the interconnect feedback regulation of the proteins in the TTFL complicate the interpretation of the correlation between their expression and diseases. Whether CRY1 could be a novel target for cancer cell metabolism needs to be further evaluated *in vivo*.

## Experimental procedures

### Cell lines and cell cultures

All cell lines in this study were maintained using the Dulbecco's modified Eagle's medium (DMEM) medium with 4.5 g/l glucose (GIBCO) supplement with 10% fetal bovine serum in a humidified at 37 °C with 5% CO_2_. When experiments require media with different glucose concentrations, glucose-free DMEM (GIBCO) was mixed with high-glucose DMEM (4.5 g/l) to change the glucose concentration in the medium. Cell lines were validated mycoplasma-free by 4′,6-diamidino-2-phenylindole staining.

The Cry1/2^−/−^, Per1/2^−/−^, Nr1d1/2^−/−^ (CPN^−/−^) (26) was used as the parental cell line to generate CPN^−/−^ CRY1 and CPN^−/−^ CRY2 cell lines. The DNA fragments of mouse Cry1 and Cry2 were PCR-amplified from the complementary DNA (cDNA) library of the PER1/2^−/−^, NR1D1/2^−/−^ (PN^−/−^) cell line (8) mouse embryonic fibroblast expressing endogenous CRY1 and CRY2. Both DNA fragments were inserted into the pWZL-blast vector by restriction enzyme digestion and ligation. Retrovirus carrying CRY1 or CRY2 genes were prepared and infected CPN^−/−^ cell line. CPN^−/−^ CRY1 and CPN^−/−^ CRY2 cell lines were selected as single colonies by blasticidin (5 μg/ml). Expression of CRY1 or CRY2 was assayed using Western blot.

The MDA-MB-231 (MB231) and MCF-7 breast cancer cell lines were purchased from the Bioresource Collection and Research Center in Taiwan. To generate MDA-MB-231 cells expressing exogenous human CRY1 (MB231+CRY1), human Cry1 cDNA was cloned from the cDNA library of HEK293T human embryonic kidney cells into pcDNA4-Myc-His vector. The vector carrying the human Cry1 gene was transfected into MB231 and cells resistant to the zeocin after transfecting this plasmid were selected as single colonies by zeocin (200 μg/ml). Expression of exogenous CRY1 could be detected in Western blot as a slower migrated band than endogenous CRY1 protein due to the fusion of molecular tags (Myc and His tags).

The method used to synchronize the MCF-7 cell line refers to the published protocol (51). Cells grown to confluent were starved in the serum-free medium for 12 h and then were shocked with the medium containing 50% fetal bovine serum for 2 h. After synchronization, cells were kept in the serum-free medium at 37 °C and collected at different times.

For the suspension culture of MB231, MB231+CRY1, and MCF-7 cell lines, we used ultra-low attachment cell culture dishes that cells could not attach (Bioinert plate, Cat. No: 81150, from ibidi GmbH).

### Measurement of lactate and glucose concentration in the cell culture medium

We used enzyme-mediated colorimetric assays to determine the lactate (ab65330, Abcam) and glucose (ab102517, Abcam) following the instructions of the products. Cell culture media (0.5% v/v) were collected at different times and stored at −80 °C until finishing the collection of all time points. Medium samples were 10-fold diluted with PBS before performing the assay to reduce the influence of other ingredients in the medium. For the lactate assay, we used the lactate standard provided by the kit to convert the absorbance of light to lactate concentration. For the glucose assay, we used the high-glucose DMEM and glucose-free DMEM to prepare the glucose standard in DMEM for conversion of the absorbance of light to lactate concentration.

### RNA sequencing analysis

Two RNA sequencing data sets were used in this study. One has been published (GSE157946) (26) with mRNA sequencing data from CPN^−/−^ and PN^−/−^ cell lines. Another data set was generated in this study containing mRNA sequencing data from CPN^−/−^, CPN^−/−^ CRY1, and CPN^−/−^ CRY2 cell lines. Total RNA from these cells was prepared when the cells were around 80% confluent using Trizol RNA reagent (Zymo Research) and the Direct-zol RNA Mini Kit (Zymo Research) following the instructions. PolyA-RNA was enriched, and the DNA library was generated using TruSeq stranded mRNA preparation kit (Illumina) and sequenced using Illumina HiSeq 4000 (paired-end, 150 bp) in Genomics. Two independent RNA preparations for each cell line were sequenced.

Sequences were mapped to the mouse genome (mm10) using RNA STAR (Galaxy 2.6.0b-1) (53). The numbers of mapped reads of each gene were counted using featureCounts (Galaxy 1.6.0.6) (54). Counts of sequencing read were further used for differential gene expression analysis using Deseq2 (1.22.2) (55) in the R program (https://www.r-project.org/). Log2FoldChange compared to CPN^−/−^ represents the difference in gene expression. Normalized read counts mapped to different genes (baseMean) were a reference of relative expression levels between genes in all samples. To visualize the sequencing results, mapped reads were separated into two strands, normalized to the sequencing depth and then converted into the file for genome browsers as mentioned in the previous research (26).

For pathway analysis, R packages “fgsea” (56) for GSEA and “clusterProfiler” (57) were used. The mouse version of gene sets of hallmark pathways from the Molecular Signature Database was downloaded from the website of the bioinformatics service at Walter and Eliza Hall Institutes of Medical Research.

### ChIP and real-time PCR

Total RNA was purified using the DirectZol RNA Miniprep kit (Zymo Research). RNA concentration was determined by Qubit RNA HS kit (Thermo Fisher Scientific). cDNA was prepared from the same amount of total RNA using the PrimeScript RT Reagent kit (Takara Bio). Real-time PCR analysis was performed using iQ SYBR Green Supermix and CFX96 Real-Time PCR Detection System (Bio-Rad Laboratories). The relative levels of genes were first normalized to the internal control gene, Gapdh, and then compared to the reference cell line (CPN^−/−^ or MDA-MB-231).

The ChIP experiment followed the same protocol in our previous study with minor modifications (8) due to the different sonicators. Instead of the radioimmunoprecipitation assay buffer in the previous protocol, 1% SDS was used for DNA fragmentation by sonication using Biorupter (Diagenode). BMAL1 antibody (Bethyl) and the CRY1 antibody (7) were used to pull-down DNA fragments bound by BMAL1 or CRY1. Rabbit immunoglobulin G and rat immunoglobulin M were used as the isotype controls of BMAL1 and CRY1 antibodies in the immunoprecipitation step, respectively. DNA fragments collected before and after immunoprecipitation were quantified using real-time PCR after reverse-crosslinking and protein removal.

Sequences of primers for quantification of RNA and DNA levels using real-time PCR in this study are provided in the supporting information (Table S4).

### Antibodies and Western blot

Cells were either collected from the plate by scraping (adhesion culture) or from the culture medium by centrifugation (suspension culture). After PBS wash, cells were lysed with radioimmunoprecipitation assay buffer (50 mM Tris-Cl pH 8.0150 mM NaCl, 1 mM EDTA, 1% Triton X-100, 0.1% SDS, and 0.1% sodium deoxycholate). After centrifugation (15,000*g* for 15 min), supernatants were collected, and the protein concentrations of lysates were determined by the QuantiChrom Total Protein Assay Kit (BioAssay System). Equal amounts of proteins from samples were separated by SDS-PAGE, and the target proteins were detected using Western blot. The Western blot results were collected using the ChemiDoc Imaging System (Bio-Rad Laboratories) and quantified by the Image Lab software (Bio-Rad Laboratories). Signals were first normalized to the loading control (ACTIN) and then expressed as the relative levels to reference cell line.

The antibody for detecting mouse CRY1 was described (26). Antibodies for the detection of mouse and human BMAL1 (Bethyl Laboratories, A302-616A), human CRY1 (Bethyl Laboratories, A302-614A), mouse and human CRY2 (Invitrogen, MA5-37755), human PER1 (Genetex, GTX128966), human PER2 (Genetex, GTX129688), mouse and human PDK1 (Abcam, ab202468), human PDHA Ser293 phosphorylation (EMD Millipore, ABS204), and ACTIN (Genetex, GTX629630) were from commercial sources. The specificity of the antibodies was evaluated by the detection of a single band at the expected molecular weight of the target protein.

## Data availability

The mRNA-Seq datasets from CPN^−/−^, CPN^−/−^ CRY1, and CPN^−/−^ CRY2 cell lines in this study can be found in the Gene Expression Omnibus (GEO) repository. https://www.ncbi.nlm.nih.gov/geo/query/acc.cgi?acc=GSE246853.

## Supporting information

This article contains supporting information.

## Conflict of interest

The authors declare that they have no conflicts of interest with the contents of this article.

## Figures and Tables

**Supplemental Figure S2:**