# Supplementary material for: Circadian modulation of glucose utilization via CRY1-mediated repression of Pdk1 expression
Source: J Biol Chem. 2024 Jan 8;300(2):105637. doi: 10.1016/j.jbc.2024.105637 (PMC10869264; doi:10.1016/j.jbc.2024.105637)
Supplement: Supplemental Tables and Figures Legend [file mmc1.docx]

**Table S1: Relative gene expression in PN^-/-^, CPN^-/-^ CRY1and CPN^-/-^ CRY2 cell lines compared to CPN^-/-^ CRY1 cell line**

Relative mRNA levels of 27,179 annotated genes in PN^-/-^, CPN^-/-^ CRY1, and CPN^-/-^ CRY2 cell lines compared to CPN^-/-^ CRY1 cell line were provided. “NA” indicates no reads mapped to the gene in the dataset. Relative mRNA levels and statistical significance are expressed as “log2FoldChange” and “svalue”. Genes overlapped with the BMAL1-ChIP-seq (GSE93318) are marked as “YES” in the last column.

**Table S2: Pathways affected by CRY1 and CRY2 by Gene-Set-Enrichment analysis (GSEA)**

Hallmark pathways with significant differences between CPN^-/-^ CRY1 and CPN^-/-^ cell lines or between CPN^-/-^ CRY2 and CPN^-/-^ cell lines were analyzed by Gene Set Enrichment analysis. The enrichment score (ES) and the adjusted p-value (padj) of both comparisons are provided.

**Table S3: Pathways affected by CRY1 and CRY2 by KEGG Enrichment analysis**

Genes showed statistically significant differences in expression between CPN^-/-^ CRY1 and CPN^-/-^ cell lines or between CPN^-/-^ CRY2 and CPN^-/-^ cell lines were analyzed by KEGG enrichment analysis. The ratio of differentially expressed genes in specific pathways (Gene Ratio) and the adjusted p-value (padj) of both comparisons are provided.

**Table S4: Primers used in real-time PCR**

Sequences of primers used in the real-time PCR to quantify DNA fragments from ChIP experiments and cDNA fragments from RNA are provided. The first character in the symbol represents the species. For example, mPDK1 represents the primer set for mouse PDK1 RNA. Symbols with “_P” represent primer sets for amplifying the regulatory region of genes in the ChIP experiments.

**Figure S1: Comparison of CRY-affected genes in different RNA-seq datasets.**

Numbers of genes in different CRY-expressing cell lines with statistically significant differences (svalue < 0.05) in the mRNA levels compared to CPN^-/-^ cell line are shown in black circles. CRY-affected genes were divided into a “lower” group and a “higher” group. Numbers in the parentheses are numbers of genes overlapped with the BMAL1 binding sites according to BMAL-ChIP-seq data. Numbers of genes with statistically significant differences in two comparisons and in the same group were shown between the two black circles. Numbers of genes with statistically significant differences in all comparisons and in the same group were shown in the grey circle in the center. Ten genes with lower mRNA levels in all CRY-expressing cell lines compared to CPN^-/-^ cell line and overlapped with BMAL1-binding sites were listed and highlighted in red.

**Figure S2: Analysis of CRY1, PER2, PDK1 protein levels and PDH phosphorylation of MCF-7 cell line.** (A) Western blot results of PDK1, PDH phosphorylation (PDHA Ser293p), PER2 and CRY1 in the MCF-7 cell line grown in adhesion (Adh.) or suspension (Sus.) are shown. ACTIN served as the loading control. Triplicates (#1, #2 and #3) were collected and analyzed. (B) Western blot results of PDK1, PER2 and CRY1 in the MCF-7 cell line after synchronization by serum shock.
